# Recent explosive lava-water interaction in Tharsis, Mars

**DOI:** 10.1038/s44453-026-00031-2

**Published:** 2026-04-02

**Authors:** Bartosz Pieterek, Thomas J. Jones

**Affiliations:** 1 Geohazard Research Unit, Institute of Geology, Adam Mickiewicz University in Poznan, Poznan, Poland; 2 Lancaster Environment Centre, Lancaster University, Lancaster, UK

**Keywords:** Planetary science, Solid Earth sciences

## Abstract

The spatiotemporal distribution of water on Mars is fundamental to understanding the planet’s evolution. Comprehensive integration and analysis of high-resolution remotely sensed datasets have enabled the investigation of previously uninterpreted small-scale volcanic landforms that imply the involvement of water during formation. Although volcanism has played a dominant role in shaping the Martian surface, irrespective of the physical state of water, there is little evidence of magma-water or lava-water interactions, especially in young, late Amazonian-aged volcanic provinces. Here, by combining surface imagery with topographic data and spectral analyses, we report the presence of rootless volcanic cones formed by explosive lava-water interaction during the late Amazonian (<215 Ma). They are found adjacent to Ascraeus Mons in Tharsis and are associated with spectrally identified hydrated minerals, most likely sulfates, indicating past hydrothermal circulation potentially sustained by lava-water interactions. We contend that these young, small landforms, formed through phreatomagmatic eruptions, can help reconstruct Martian paleoclimate, identifying water ice zones, and should be considered prime targets in the future search for life, as they fulfill key habitability criteria.

## Introduction

In active volcanic regions on terrestrial planets, the interaction of magma with either underground or surface water or ice is known to trigger phreatomagmatic eruptions^1,2^. Such interaction can vary greatly in explosivity ranging from highly explosive events such as the explosive Hunga Tonga-Hunga Ha’apai eruption that experienced extensive magma-water integration^3^ and injected large amounts of water vapor into the atmosphere^4^, to predominantly effusive lava interactions^5,6^. When advancing lava flows propagate over water-logged sediments or ground ice, they can cause melting and the generation of water vapor, which triggers explosions excavating overlying parts of the lava flow. These events produce distinctive surface landforms known as rootless cones^7–10^. These conical features on top of lava thus directly imply underlying groundwater or ice at the time of their formation. Such interactions can also sustain the development of volcanically driven hydrothermal systems^1,11^, which have the potential to host diverse forms of life^12^ and later yield fossil hydrothermal deposits with valuable metal-bearing mineralization, that could be considered for future *in-situ* utilization^13^.

Given the volcanic- and water-driven geological history of Mars, such eruptions should be widely identifiable^14,15^. To date, however, lava-water interactions have been reported primarily at specific locations where fluvial or glacial landforms co-occur with volcanic activity^1,2,16–21^, mostly near the Martian dichotomy. The inferred rootless cones typically occur in clusters atop of a single lava unit and comprise similarly sized, near-circular edifices featuring well-developed central crater with a partially or fully preserved rim^1,2^. However, comparable landforms have not yet been confirmed within the Tharsis region itself, the largest volcanic province on Mars. This absence complicates current interpretations of past ice distribution^22^ and challenges our understanding of the spatiotemporal interactions between volcanism and water—processes that are fundamental for constraining past climate change, volatile stability, and the development of hydrothermal systems, which have the potential to host biosignatures^23^.

The continuous acquisition of satellite datasets and orbital camera development has increased the availability of high-resolution remotely sensed datasets that continue to reveal small-scale volcanic landforms^24^. Due to the small size of landforms formed by the lava-water interactions (ranging from tens to hundreds of meters in diameter and only several meters in height) and morphological similarity to impact craters and other topographically positive pitted landforms^15,20^, rootless cones remain challenging to accurately recognize. As a result, their identification has often been overlooked, thereby limiting a complete view of the spatial extent and timing of recent magma–water interaction on Mars. However, their recognition from orbit and morphological characterization can be greatly improved through the implementation of high-resolution digital elevation models (DEMs) produced from stereo image pairs or by applying convolutional neural network (CNN) techniques^25^. Although these spatially limited topographic datasets typically cover only fragments of regions hosting from several to dozens of small-scale landforms, analogical reasoning allows interpretations to be extended to hundreds or even thousands of similar-looking features in adjacent terrains^16^. Therefore, accurate interpretation of landform origin can only be achieved when combined with the regional geological context.

Accordingly, Tharsis, the largest volcanic province on Mars, features widespread, relatively young (<300 Ma; Late Amazonian) volcanic activity, and thus constitutes a natural laboratory with a unique assemblage of well-preserved lava flows and volcanic edifices^26–28^, offering insights into recent volcanic evolution. The volcanic complexity of this province is evidenced by the co-occurrence of features reflecting different eruptive styles^15^, including dominantly effusive low shield volcanoes^29^ and associated lava flows, as well as explosively derived cones^27,30–32^ and spatter ramparts^24,33^ attributed to lava fountaining. Specifically, the Tharsis Montes volcanoes comprise two contrasting sides, the west-northwest flanks exhibit fan-shaped glacial deposits^34–38^, whereas the opposite flanks have surfaces dominated by volcanic eruption centers and associated lava flows^29,39,40^. For the previously glaciated Tharsis Montes flanks, volcano-ice interactions have been inferred though the recognition of glaciovolcanic landforms formed under subglacial conditions^41,42^. However, the volcanically dominated south- and southeast Tharsis Montes flanks are thought to have remained ice-free, or any volcano-ice interactions evidence may have been overprinted by the youngest volcanic activity.

**Fig. 1:**
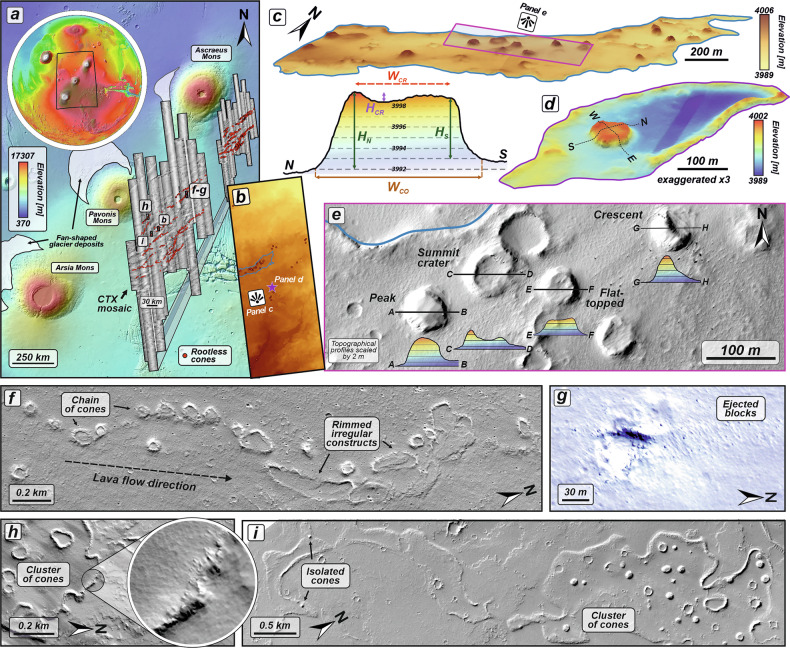
Lava flows characteristics with superimposed rootless cones. **a** Topographic map showing the locations of the documented rootless cones (red dots) south of Ascraeus Mons, one of the three Tharsis Montes volcanoes. The basemap was produced using the MOLA shaded relief basemap (463 m px^−1^) overlaid by the MOLA- High Resolution Stereo Camera (HRSC) DEM^106^ (200 m px^−1^). The CTX-based mosaic presents the area with identified rootless cones. The black rectangles on the CTX images mark the location of the High Resolution Imaging Science Experiment (HiRISE) images presented in other panels. **b** HiRISE-derived DEM with marked rootless cones, generated from stereo-pair images HI_033961_1870 and HI_034462_1870. The blue solid line outlines an individual lava flow with rootless cones on top. The violet star marks the location of the rootless cone featured in panel (**d**). **c** Oblique 3D view of the lava flow with superimposed rootless cones. The terrain model was produced from the HiRISE-derived DEM. **d** Example of an isolated rootless cone, with the topographic profile lines used for morphometric measurements. The corresponding N-S topographic profile illustrates the measurements made in this study. The abbreviations correspond to basal width (*W*_*CO*_), summit crater width (*W*_*CR*_), depth of the summit crater (*H*_*CR*_), heights of the cone measured on both profile sides (*H*_*N*_ and *H*_*S*_), respectively. **e** HiRISE image (ESP_033961_1870) showing a cluster of rootless cones with different morphologies. **f** Chain of rootless cones aligned along the direction of the lava flow from HiRISE image ESP_034818_1890, centered at 8.753°N and 260.008°E. **g** Semi-circular depression with an elevated rim, associated with dispersed fragmented lava blocks. The view is derived from the infrared-red-blue (IRB) color HiRISE image ESP_034884_1890, centered at 8.781°N and 260.003°E. **h** Rootless cone cluster located proximal to the elevated flow margins (shown in inset). **i** Another rootless cone cluster. The view is derived from the HiRISE image ESP_038062_1870, centered at 6.8643°N and 258.325°E.

Here, we conducted a dedicated mapping campaign focused on identifying small-scale mounds south of the Ascraeus Mons volcano (Fig. 1). Through detailed morphological and spectral analyses using high-resolution images from the High-Resolution Imaging Science Experiment^43^ (HiRISE) and hyperspectral datasets from the Compact Reconnaissance Imaging Spectrometer for Mars^44^ (CRISM), we discovered rootless cones—direct products of lava-water interaction. Their relative stratigraphy, supplemented by crater counting, constrains their young formation age and allows us to document the distribution of subsurface water or ice within Tharsis during the recent geological past. Our observations also provide evidence for fossilled hydrothermal activity and consequently, such locations should be considered prime target in the search of life, as they met key habitability requirements in the past—namely, the presence of liquid water and energy from the volcanic heat^12,45^.

## Results and discussion

### Geomorphology and stratigraphy

To the south of Ascraeus Mons, lava flows form an extensive field that is slightly inclined toward the NNE, enabling lava to advance into the surrounding regions. We find conical edifices directly atop these lava flows (with at least 2114 cones/edifices identified; Supplementary Data 1), which we interpret here as rootless cones. Their origin is explained further in the first discussion section of this contribution. They often form clusters near the flow fronts or margins, or are aligned in flow-parallel chains, however, isolated features do also occur (Fig. 1). These conical edifices comprise morphologically distinct assemblages characterized by a central depression (crater), a flat-topped summit, or a central peak. In some cases, the central depressions have a semi-circular or irregular shape and are surrounded by a raised rim (Fig. 1). The close proximity of individual edifices can sometimes lead to spatial overlap, modifying their conical shape. Moreover, their putative explosive origin occasionally produces crescent-shaped structures and a wide field of fragmented blocks.

HiRISE-based measurements document a large, relatively homogenous edifice population (Supplementary Data 1) with an average basal width of 96 ± 31 m (1 SD; *n* = 249) and a crater width of 43 ± 18 m (1 SD; *n* = 207), yielding a width ratio of 0.42 ± 0.11 (1 SD; *n* = 207). Ratios between perpendicularly measured basal widths yield an average edifice circularity of 1.00 ± 0.17 (1 SD; *n* = 218). Stereo-pair-derived DEMs reveal that these edifices have an average height of 3.8 ± 2.0 m (1 SD; *n* = 178), resulting in average slope values of 7.4 ± 3.9° (1 SD; *n* = 170). The average depth of the central crater, when present, is 1.2 ± 0.8 m (1 SD; *n* = 133).

The edifice clustering and their preferential occurrence on top of lava flows may imply a relationship between their formation and the lava thickness. Although pre-eruption topographic elevation data are unavailable for Mars, careful analysis of HiRISE imagery and the corresponding DEM reveals isolated patches of lower terrains that preserve the pre-eruption surface—allowing us to determine their elevation and model the pre-eruption surface and thus the spatial distribution of lava thickness (Figure S1). At locations where the edifices are superimposed, lava thickness ranges from 9.8 to 25.2 m, with an average of 18.4 ± 3.4 m (1 SD; *n* = 118).

**Fig. 2:**
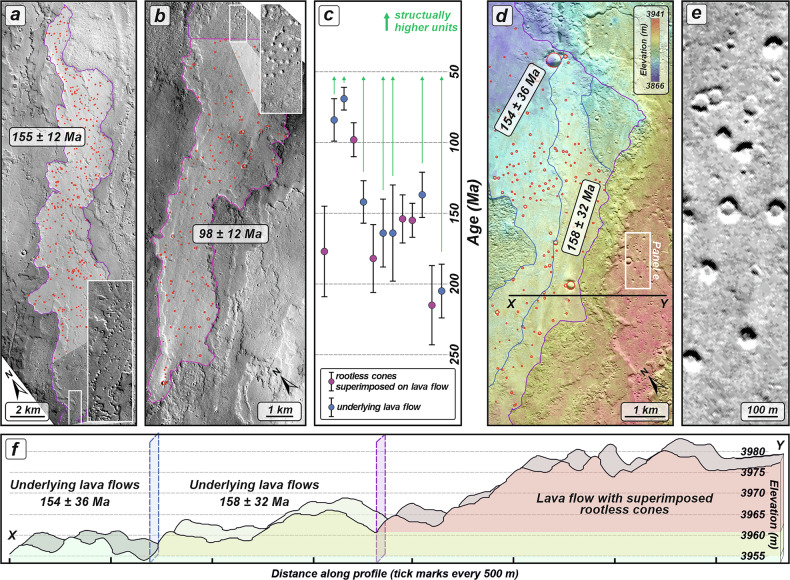
Compilation of the age-dating results constraining the formation age of the rootless cones. **a**–**b** Examples of lava flows with superimposed rootless cones. Parts of the lava flows that lack rootless features were selected for crater counting to avoid biasing the obtained age with pseudocraters formed during phreatic eruptions. Insets show close-up images of the rootless cones. The images comprise CTX images: **a** D05_029069_1867, centered at 6.667°N and 259.696°E, and (**b**) U23_079800_1861, centered at 6.118°N and 259.1998°E. Violet solid lines outline the areas used for crater counting, and red circles mark the locations of the mapped impact craters. **c** Plot showing age-dating results. The structurally lower lava flows predate the flows with rootless cones, representing the maximum possible age of the phreatomagmatic eruptions. Consequently, the structurally higher flows must be younger. Green arrows indicate that the rootless cones are geologically younger than the age determined for the underlying lava flow. **d** Visualization of the structurally lower lava flows (outlined by blue solid lines), which predate emplacement of the uppermost lava flow bearing superimposed rootless cones. The white rectangle marks the area shown in the close-up image in panel (**e**), while the black solid line indicates the location of the topographic profile shown in panel (**f**). The basemap is a CTX-derived stereo-pair DEM generated by MarsSI, using P14_006600_1881 and D22_035596_1877, centered at 7.675 and 258.088°E. **e** Close-up image of the rootless cones (CTX image U11_075435_1866). **f** Corresponding topographic profile based on the CTX-derived DEM, showing the structural relationship between lava flows. Blue and violet dashed vertical panels correspond to the unit outlines shown in panel (**d**).

The ages of the individual lava flows upon which the edifices are directly superimposed represent the best approximation of the cone formation age. Age-dating results for the edifice-hosting lava flows range from 215 ± 28 to 98 ± 12 Ma (Table S1), whereas underlying lava flows that predate those with superimposed edifices yield ages ranging from 205 ± 19 to 69 ± 8 Ma, indicating the maximum possible ages for the emplacement (Fig. 2), which corresponds to the late Amazonian epoch.

### Spectral data

**Fig. 3:**
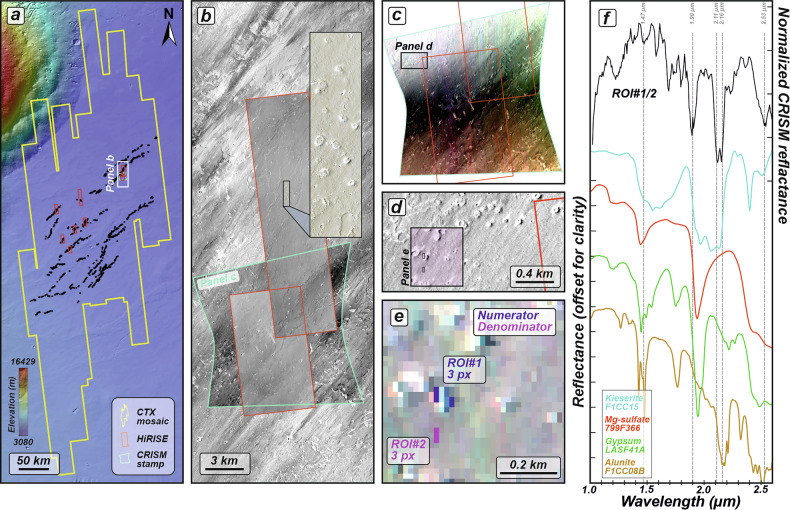
Spectral evidence for fossil hydrothermal systems associated with rootless cones. **a** Topographical context map of the investigated area south of Ascraeus Mons, showing the locations of datasets used in this figure. The basemap was produced using the MOLA shaded relief basemap (463 m px^−1^) overlaid by the MOLA-HRSC DEM^106^ (200 m px^−1^). **b** Compilation of the datasets used for spectral analyses. Analyses were conducted using the Compact Reconnaissance Imaging Spectrometer for Mars (CRISM; 18 m px^−1^), outlined by turquoise solid line, whereas cones were visualized by HiRISE images (0.5 m px^−1^), outlined by red solid lines. The outline colors correspond to those in (**a**) and following panels. The black rectangle presents zoomed-in image of the rootless cones of the infrared-red-blue (IRB) color HiRISE image ESP_034818_1890, centered at 8.7525°N and 260.0035°E. The violet star marks the location of the HiRISE-based close-up image shown in the inset at the bottom right. **c** CRISM stamp (FRT00016E46, centered at 8.6099°N and 259.9877°E) used for spectral analyses. **d** Close-up CTX image showing a cluster of rootless cones targeted by the CRISM stamp. The violet rectangle indicates the area where regions of interest (ROIs) for spectral analysis were selected. **e** Corresponding zoomed-in view of the CRISM stamp showing the locations of ROI#1 (numerator) and spectrally neutral reference (denominator; ROI#2). Numbers correspond to the spectra in (**f**). **f** Compilation of the normalized CRISM spectrum for ROI#1 compared with laboratory-derived sulfate spectra^46^, including kieserite (F1CC15), Mg-sulfate (799F366), gypsum (LASF41A), and alunite (F1CC08B). The normalized spectrum of ROI#1 is scaled to the right vertical axis (values divided by 0.1). Reference spectra are scaled to the left vertical axis (also divided by 0.1). Spectra are vertically offset for clarity. The horizontal axis ranges from 1 to 2.6 µm.

Only one CRISM targeted image (ground pixel resolution of 18 m px^−1^) covers a portion of the studied edifice cluster. The infrared (IR)–derived browse products (HYS and PHY) indicate that some edifice locations exhibit spectral characteristics consistent with hydrated minerals, including possible sulfates, carbonates, or hydrated silica, although these browse products are not uniquely diagnostic (Figure S2a–c). The HYD browse product, which is sensitive to hydration-related absorption, highlights several pixels associated with the edifice that exhibit strong hydration signatures (Figure S2d). In particular, a CRISM spectrum extracted from a region of interest (ROI) located on an edifice flank was normalized to spectrally neutral terrain within the same detector column. The resulting normalized spectrum exhibits strong absorption features at ~1.90 and ~2.16 µm, along with a downturned spectral slope associated with the absorption feature centered at ~2.52 µm (Fig. 3 and S3). The absorption feature centered at ~1.90 µm is consistent with the presence of hydrated or hydroxylated minerals within ROI#1. The normalized CRISM spectrum (Fig. 3) shows similarities to laboratory spectra of sulfate-bearing minerals^46^ and, in particular, exhibits an absorption at ~2.17 µm that is consistent with alunite-like features. However, the spectrum lacks a diagnostic combination of the accompanying absorption bands required to confidently assign a specific sulfate phase. Accordingly, we interpret this detection as a sulfate–bearing, hydrated mineral, rather than a definitive identification of a specific sulfate mineral.

### Explosive lava-ice interactions

**Fig. 4:**
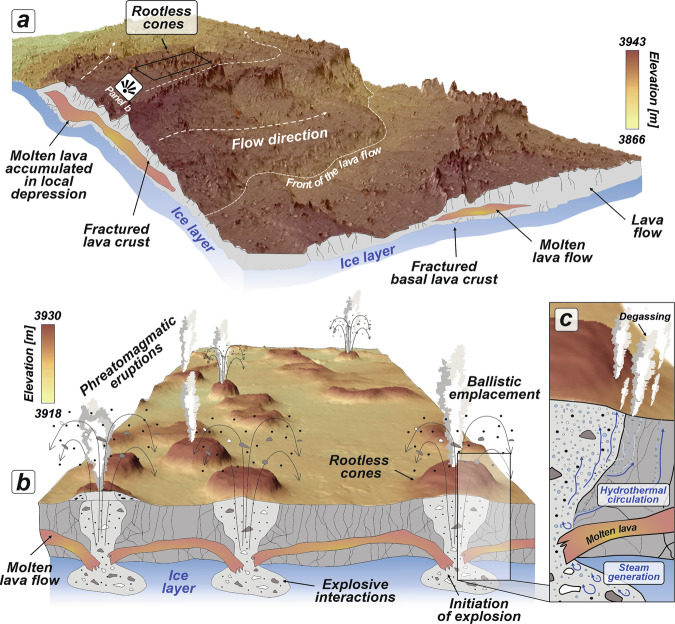
Conceptual model illustrating phreatomagmatic eruptions in Tharsis, resulting in hydrothermal circulation. **a** 3D view of a lava flow advancing over an ice-rich layer, with flow direction indicated by white dashed arrows. Cross-sections of the model depict a partially solidified lava flow, showing a solid crust both on the surface and at the base, enclosing a molten lava core. The surface terrain is derived from HiRISE DEM data (stereo pair: HI_034172_1880 and HI_035596_1880) and vertically exaggerated 30 times. **b** Schematic representation of the inferred process leading to rootless eruptions. The molten core of the lava flow concentrates in local topographic depression, heats the underlying ice-rich layer, causing steam generation, and consequently, triggers explosive phreatomagmatic eruptions. **c** Close-up view of the associated hydrothermal system powered by the lava-water ice interactions, leading to the formation of hydrated minerals.

We hypothesize that the previously unreported small-scale, conical-shaped features south of Ascraeus Mons are the direct products of lava–ice interactions, termed rootless cones (Fig. 4). Their structural relationship, in which edifices are superimposed on lava flows spreading from the south towards northeast of Ascraeus Mons (Fig. 1a), resembles both terrestrial^9,47,48^ and Martian rootless volcanic constructs^1,16,18,19,21,49–53^, also known as pseudocraters, which form through explosive phreatomagmatic eruptions. Their exclusive superposition on the surfaces of individual lava flows suggests that their formation was strictly controlled by, and limited to, lava flow emplacement. This challenges any direct connection between the cones and subsurface feeder dykes, and is a diagnostic feature of rootless cones^1,2^. Our understanding of lava-ice interactions and their products, however, may be biased by the fact that the structurally youngest lava flows, which almost completely resurfaced these terrains^28,54^, could have buried almost every remnant of previous ice-related landforms. Our findings enable a more accurate spatiotemporal reconstruction of ice distribution at the time of the associated volcanic activity.

However, previous studies have also proposed that morphologically similar features, particularly in the northern lowlands, may form through a variety of processes^15,20^. In the geological context of Tharsis, sedimentary volcanism in the form of mud volcanoes can be excluded as a potential explanation for the observed cones. Such volcanism requires the accumulation of relatively thick, water-logged sedimentary deposits that become over pressurized, thereby triggering fluid expulsion^55,56^. Consequently, sedimentary volcanism is restricted to specific locations on Mars where favorable conditions were present in the past^57–60^. By analogy, interpretations invoking periglacial landform formation, such as pingos, which are produced by ground-ice growth, are also highly unlikely. The studied cones are situated atop effusive lava flows that promote drainage rather than water retention or sediment accumulation, thereby inhibiting the ice-lens growth necessary for pingo formation. Moreover, such periglacial landforms are typically accompanied by radial extensional cracks^61^ related to ice growth or subsequent melting, which contrasts with the well-developed, symmetrical summit craters of the studied cones. Therefore, an igneous volcanic origin is the most plausible within the given geological context. On Earth, although springs and geysers—which can construct conical landforms—are not exclusively related to volcanic terrains, volcanic activity strongly favors their development. In both cases, they require elevated subsurface temperatures associated with shallow magmatic intrusions or plumbing systems, as well as an abundant groundwater supply that can sustain their activity and subsequent cone construction. Such origin is also unlikely for our cones, as lava flows are unable to either develop a stable aquifer or to deliver sufficient heat over extended periods of time to sustain these systems.

On volcanic terrains, morphologically similar volcanic structures, known as hornitos, can form through outgassing-driven spattering from an active lava flow^62^. These processes create small, irregular mounds with rough surface textures, typically composed of agglutinated spatter deposits and lack well-defined summit craters^63^. This origin is unlikely in our case, as there is no evidence of cone-associated lava flows—features commonly present when welded spatter transitions into rheomorphic flows, or when lava overspills from the hornito and spreads onto adjacent terrain. Moreover, when a lava tube system is active, internal pressure increases and increased lava fluxes can lead to the formation of dome-shaped structures on the lava flow surface known as tumuli^29^. This origin is again unlikely for our identified edifices. Tumuli do not form central craters due to the absence of explosive activity and are instead mostly characterized by a domed surface with cracks. Therefore, we infer that these late Amazonian-aged lava flows, likely originated from the distributed volcanism of Ascraeus Mons, and advanced over an area locally containing surface ice or ice-saturated ground leading to explosive phreatomagmatic events forming the rootless cones observed.

**Fig. 5:**
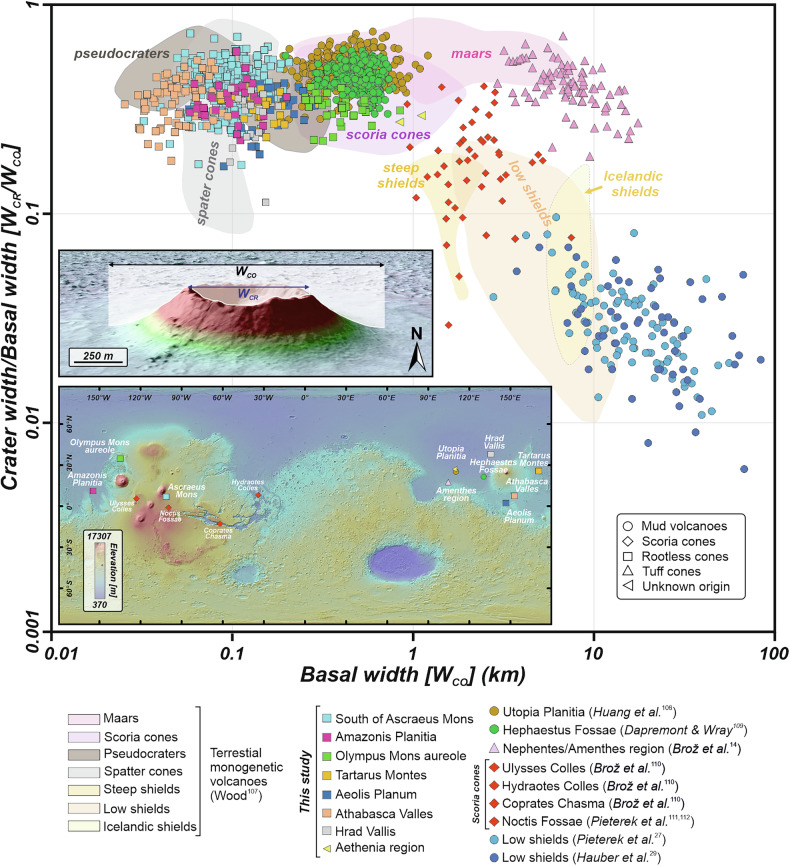
Morphometric comparison of distinct Martian fields hosting small-scale conical edifices. The plot shows the compilation of the summit crater width/basal width (*W*_*CR*_*/W*_*CO*_) ratio versus the basal width, *W*_*CO*_. Morphometric results are compared along with various types of terrestrial monogenetic volcanoes^107^. For most Martian fields, we conducted dedicated measurement campaigns using HiRISE images, HiRISE-derived DEMs, or a combination of both. Inset shows a 3D visualization example of a rootless cone located in Aeolis Planum, with a illustration of the measured parameters. The morphometric data clearly show that the cones documented south of Ascraeus Mons overlap with other known rootless cone populations on Mars^1,16,19,50,52,53^. The locations of the fields included in this analysis are shown on a topographic map derived from MOLA–HRSC data (200 m px^−1^), underlaid by the MOLA shaded relief basemap (463 m px^−1^). The datasets used for this morphometric comparison include results for mud volcanoes^108^^,109^, tuff rings^14^, scoria cones^110^^,111,112^, and low shield volcanoes^27^^,29^.

The morphological similarity (Figs. 5 and S4) of the studied cones to other interpreted rootless constructs across Mars^1,2,21,50^ also supports the interpreted phreatomagmatic origin. Morphometric comparison shows that our cones share similar parameters with the other Martian rootless cones previously identified (Fig. 5). Based on our manual measurements of morphometric parameters using HiRISE-derived DEMs for other locations interpreted as occurrences of rootless cones on Mars (Table S2), we found that these features are characterized by a range of sizes; however, they are clearly morphologically distinguishable from mud volcanoes and scoria cones (Fig. 5), which form larger and higher edifices^60–62^. Our cones show morphological overlap with rootless cones from Amazonis Planitia^2,19^ and Hrad Vallis^16^, which have mean basal diameters of 104.2 ± 30.5 m (1 SD; *n* = 34) and 131.1 ± 22.8 m (1 SD; *n* = 20), respectively, as well as mean crater widths of 37.1 ± 13.4 m and 38.5 ± 11.9 m. These measurements yield average crater-width-to-base-width ratios of 0.36 and 0.29, respectively. The average height of Amazonis Planitia cones is 8.0 ± 2.7 m, which is approximately twice that of our cones, whereas cones in Hrad Vallis exhibit comparable heights, with a mean value of 5.36 ± 2.1 m. Slightly larger and higher edifices of rootless-cone origin have been reported in Aeolis Planum^50^ and Tartarus region^1,18^. These cones exhibit similar morphometric parameters, with average cone diameters of 167.9 ± 53.6 m (1 SD; *n* = 30) and 145.0 ± 41.6 m (*n* = 30), as well as crater widths of 54.4 ± 23.9 m and 47.4 ± 13.7 m, respectively. Moreover, they have comparable average heights of 19.9 and 14.2 m, respectively. The largest rootless cones have been identified north of Olympus Mons^64^, with a basal width of 392.4 ± 123.1 m, a crater width of 124.5 ± 39.1 m, and a height of 28.7 ± 10.8 m. Altogether, these qualitative and quantitative morphological comparisons of small-scale conical landforms across Mars indicate that our newly documented cones located south of Ascraeus Mons represent another field of Martian rootless cones (Fig. 5).

As on Earth, two models of rootless eruptions on Mars may be considered, depending on the interaction between the lava flow and ice-bearing substrate. The first, the static heat model, involves rapid steam generation and subsequent phreatic explosions caused by water — specifically, ice-melted water in our case—being trapped beneath the advancing lava flow^7^. The second model assumes the dynamic mixing between the lava flow and the saturated substrate (i.e., the Martian regolith with ground ice)^2,49^. Here, due to the overlapping nature of multiple lava flows and the likely compositional similarity between the flows and the underlying Martian regolith, distinguishing evidence of dynamic mixing from remotely sensed imagery is unfeasible. However, regardless of the specific lava-water interaction mechanism, we infer that the thermal energy of the lava, flowing within preferred internal pathways such as lava tubes, would have caused subsurface ice to melt and vaporize, generating steam that triggered explosive interactions (Fig. 4). These explosions ballistically expelled both fragments of molten lava and overlying lava crust, to form cones with well-developed central craters. The chains of rootless cone observed likely follow a tube system that was active during the eruption^48^. Higher concentrations of the rootless cones near the elevated margins of the lava flows can be explained by ice/water confinement of lava at the time of emplacement (Fig. 1). This likely led to lava stagnation, allowing more prolonged interactions with the ground ice, which in turn, generated more rootless cones. A similar process was observed during the 2010 Fimmvörðuháls eruption in Iceland, where advancing lava flows were halted by a meltwater channel that had developed in front of the flows^65^.

### Insight into the spatiotemporal distribution of water ice

In addition to widespread distributed volcanism^27,66^ and associated landforms such as lava flows^26,28,40^, the Tharsis Montes exhibits fan-shaped glacial deposits on their west-northwest flanks, which are interpreted as the remnants of Amazonian-aged, cold-based tropical mountain glaciers^34–38^. These observations, coupled with crater-count data (~220 Ma for Ascraeus Mons), imply that substantial volumes of ice were present and stable at tropical, equatorial latitudes during extended time periods (~45–150 Myr^34^;), within the middle to late Amazonian^34,67,68^. The high obliquity^67^ of Mars’ spin axis, around 35–40°, led to episodic cold periods (glaciations) mostly affecting the mid-latitudes (30–60°), enabling the formation of ice- and glacier-related landforms. During episodes of even higher obliquity, such as 45°, these icy zones may have extended into equatorial regions, where water vapor condensed from the atmosphere could accumulate until the obliquity decreased^22,68,69^. Even during these high-obliquity periods when ice reached equatorial latitudes, it was preferentially concentrated on elevated topographic features such as Tharsis Montes^70^. The current obliquity of ~25°, which is lower than the long-term Amazonian average^71^, may still lead to short-duration atmospheric water vapor condensation and subsequent frost formation at the summit regions of the largest Tharsis volcanoes^72^.

Through the analysis of ice-related landforms, Zhou et al.^22^ provided evidence that a substantial amount of post-glaciation subsurface ice may have been preserved within impact craters until recently (220–140 Ma) in the lower latitudes of Mars. These observations extend the latitudinal range of ice-related features on Mars closer to the equator. Moreover, current Martian climate simulations^70^ suggest that mildly explosive eruptions during the late Amazonian period^24^, attributed to temporally concentrated distributed volcanism in Tharsis^26,27,66^, could have potentially contributed to the development of ice-rich substrates on both local and global scales^70^. The temporal overlap between the recognized explosive-origin deposits in Tharsis (<100 Ma) and our identified rootless cones may indicate that volatiles released during explosive eruptions could have been accumulated near the major Tharsis volcanoes. Consequently, syn- or post-glaciation advancing lava flows would have had abundant opportunities to interact with locally preserved ice near the Tharsis Montes. However, interestingly, to the south-southeast of the Tharsis Montes volcanoes, evidence for the presence of ice and its interaction with advancing lava flows has, until now, been lacking.

Our identification of rootless cones south of Ascraeus Mons challenges the inference that ice deposits were rare at low latitudes on Mars, particularly near the equator in late Amazonian. Although the spatial distribution of the rootless constructs extends from the southern lava apron toward the northeast (Fig. 1a), the clustering and presence of isolated cones atop the lava flows suggest a more localized occurrence of water ice rather than a continuous ice-bearing layer. Possibly, extended periods of environmental conditions favorable for water frost formation, such as those documented for Tharsis volcanoes^72^, followed by the accumulation of sufficient subsurface ice, could have enabled lava–ice interactions. Therefore, the age range of rootless cone formation (215 to <69 Ma; Fig. 2) may reflect episodic cold periods associated with successive high-obliquity intervals during late Amazonian^71^, that enabled (sub)surface ice accumulation. It may also simply reflect the periods of eruptive activity involving lava flow effusion^28^, or the periodic mutual occurrence of both processes. These observations reinforce models of more continuous^73^, rather than episodic and rapidly declining^74,75^, volcanic activity during the Amazonian and highlight the interaction between volcanism and ice over extended timescales. This appears to be the case for other rootless cones on Mars, however their formation ages are poorly constrained, although most are thought to have formed during the late Amazonian^1,19,51^. Our age estimates are consistent with those of other Martian rootless cones (<100 Ma in Amazonis Planitia^19^, 75–250 Ma for Tartarus Colles^1^, 35–140 Ma in Marte Vallis^76^, and <10 Ma in Athabasca Valles^51^) and appear to partly overlap with the documented^34,67^ low-to mid-latitude glaciation events that occurred between 700 and 100 Ma.

Although crater-count ages overlap within the uncertainties (Fig. 2). Such age equivalence does not preclude substantial changes in environmental conditions at the time of lava emplacement, particularly given the limited temporal resolution of crater dating relative to climatic processes, for example, associated with Mars obliquity^71^. For example, the lava flows that host rootless cones might have been emplaced during or shortly after an interval of enhanced ice deposition, potentially driven by higher-obliquity conditions. In contrast, earlier lava flows may have erupted under comparatively drier substrate conditions, insufficient to generate explosive lava–ice interactions. Moreover, differences in lava emplacement dynamics (e.g., higher effusion rates or longer eruptions) may also have controlled the triggering of phreatomagmatic eruptions. Finally, it is also possible that rootless cones may have formed during earlier eruptive phases but were subsequently buried by later lava emplacement.

The ages obtained in this study largely overlap with published age constraints for rootless volcanic constructs on Mars^1,16,18,19,21,49–53^, reinforcing the interpretation that subsurface ice or ice-saturated sediments remained available for lava–ice interactions well into the late Amazonian. Notably, rootless cone fields reported previously are generally located at moderate elevations (e.g., Elysium and Amazonis Planitia), whereas the cones examined here are the highest-elevation examples documented to date (Fig. 5). This observation implies that ice was capable of persisting across a broader range of environmental conditions than previously recognized. The apparent clustering of rootless cones in mid- to lowland terrains may therefore partly reflect preservation or detection biases.

Moreover, the presence of rootless cones at high elevations, and thus relatively low atmospheric pressure, within the Tharsis region suggests that late Amazonian lava flows retained sufficient thermal energy and volume, reflected by their lengths and relatively large thicknesses^28^, to trigger explosive lava-ice interactions. Given that ice stability is dependent on surface temperature and atmospheric pressure^77^, our findings imply that late Amazonian atmospheric conditions, potentially involving higher mean atmospheric pressures or episodic high obliquity phases, intermittently permitted the persistence of (sub)surface ice. Therefore, our results provide important constraints on the distribution of ground ice, which should be accounted for in estimates of the spatiotemporal distribution of subsurface ice on Mars and, consequently, in climate reconstructions.

### Phreatomagmatic-induced hydrothermal activity

Lava-ice interactions are one of the ways that hydrothermal activity can be initiated, especially within active volcanic areas^9,78^. Lava-induced heating likely causes steam generation^79^, which can circulate through cracks in fractured lava (Fig. 4), thereby creating hydrothermal circulation. The heated water, either in liquid or vapor state, rises towards the surface, leaching sulfur from the lava or incorporating it from volcanic gases (e.g., hydrogen sulfide, sulfur dioxide), producing sulphate-rich hydrothermal solutions, that can then precipitate sulfate minerals either near subsurface or on the Martian surface^80^. Therefore, the hydrated minerals spectrally resembling sulfates that were identified on the rootless cone flank indicate the presence of a past hydrothermal system southeast of Ascraeus Mons. Similarly aged hydrothermal deposits, such as sulfates (both mono- and polyhydrated) and opaline silica, have been identified in Coprates Chasma and interpreted as the products of past hydrothermal activity associated with igneous volcanism, likely involving low-energy explosive eruptions and the formation of scoria cones^81^.

The longevity of the hydrothermal circulation system is primarily controlled by the lava flow thickness and the fraction of ground ice^9^. Although the pre-eruption ice fraction cannot be directly reconstructed, the integration of high-resolution imagery and stereo-pair-derived topographic data allows for constraints on the thickness of the advancing lava flows. In the broad Tharsis region, the thickness of late Amazonian-age lava flows has been reported^28,82^ to range from <10 to 90 m. Here, by reconstructing the pre-eruption surface, we calculated the lava thickness at the rootless cone locations to range from 10 to 25 m (Figure S1). These values are consistent with lava flow thicknesses associated with rootless cones documented elsewhere on Mars, including ~10 m in Marte Vallis^19^, 50 m in Aeolis Planum^50^ and near Hrad Vallis^16^, and 30–60 m of Tartarus Colles^9^. The lava thicknesses documented in this study, southeast of Ascraeus Mons, indicate that ground ice must have been located no deeper than approximately 5 to 12 meters below the base of the molten lava (about half of the lava flow thickness) in order for the generated water vapor pressure to exceed the lithostatic pressure of the overlying lava crust^83^.

With these insights into lava flow thickness, thermodynamic modeling can be used to assess the duration of Martian conditions suitable for maintaining molten ice (temperatures >273 K), thereby sustaining hydrothermal circulation^9^. These calculations^9^ show that, for a lava flow thickness of ~30 m (which is close to our thickness values) and for ground ice fractions of 0.1 to 0.3, the maximum duration that ice could have remained melted is estimated to range from 140 to 315 years. However, this longevity may be overestimated, as the model does not account for convective circulation^9^, which would accelerate cooling of the system. Therefore, we infer that the Martian hydrothermal processes documented here were likely short-lived, similar to the terrestrial short-lived, lava-induced hydrothermal systems^9,78^, and would have only continued until the lava cooled sufficiently or the supply of ground ice was exhausted.

### Heat and water vapor as a potential habitable environment

Martian volcanic sites with spectrally confirmed hydrothermal mineralization^81,84^ are of astrobiological interest, as they fulfill key habitability criteria^12^. Hydrothermal fluids generated during lava-ice interactions may have provided water vapor and potentially localized chemical energy; however, any such environments would have been highly transient. While these conditions could, in principle, sustain biological activity^16,78^, if present, any associated biosignatures would most plausibly be preserved through entrapment within subsequently precipitated sulfate minerals^85^.

Since the sulfur content in Martian basalts is relatively high^86^, the crystallization of lavas must have been attributed to the release of sulfur-rich gases during eruption^87^. The interaction of lava-ice-released fluids with sulfur-bearing volcanic gases (e.g., hydrogen sulfide, sulfur dioxide) could have generated acidic hydrothermal solutions, which may then have undergone oxidation reactions, chemically or, if present, biologically. These oxidized fluids, through reaction with surrounding rocks, may support the precipitation of hydrated sulfates, like those observed here in the CRISM spectra. Moreover, given the relatively young ages (ranging from 215 to 69 Ma) of our studied deposits, the volcanically driven, sulfate-bearing hydrated mineralization could represent one of the youngest occurrences recognized on Mars. Consequently, these deposits could offer comparatively favorable conditions for the preservation of any potential biosignatures that may have been trapped at the time of formation. Other Martian sulfate-bearing sites — including Meridiani Planum, Valles Marineris, and Gale and Jezero craters^88,89^ — are an order of magnitude older. Together, these constraints indicate that interpretations of Martian habitability focused exclusively on the early Noachian may be incomplete, and that younger, late Amazonian-aged hydrothermal systems located in volcanic terrains merit consideration as targets for future surface exploration.

## Methods

### Visible images and topographic measurements

Landform investigation was primarily based on the individual Context Camera (CTX) greyscale images with resolution of ~5–6 m/pixel, which were used to create a CTX-based mosaic (Table S3). The Global CTX mosaic of Mars^90^ was also used as a basemap for initial mapping and landform recognition. Although the CTX mosaic offers a seamless, global view of the planet, some data quality is lost due to image blending and stitching. In contrast, individual CTX images preserve subtle brightness variations, allowing more precise analysis of fine-scale features and surface details. These observations were complemented by enhanced false-color grayscale (RED) and IRB (infrared–blue) High-Resolution Imaging Science Experiment (HiRISE) images for detailed morphological descriptions. HiRISE images mostly have spatial resolutions of 50 cm/pixel, with some reaching up to 25 cm/pixel.

For geological context, we used a digital elevation model (DEM) at 200 m/pixel derived from the Mars Orbiter Laser Altimeter (MOLA) and the High-Resolution Stereo Camera (HRSC), along with a MOLA-derived shaded relief basemap at 463 m px^-1^. The availability of overlapping CTX and HiRISE images enabled the generation of stereo-pair-based DEMs with surface resolutions of 12 m/pixel and 1 m/pixel, respectively, using the dedicated data processing system MarsSI (Mars System of Information^91^). All image and data analyses associated with mapping were conducted using ArcMap (version 10.8), whereas 3D terrain models were generated in ArcScene.

Morphological parameters of the analyzed edifices were determined manually by constructing two perpendicular topographic profiles (N–S and W–E) across each edifice using the 3D Analyst toolbox in ArcMap, based on both imagery and HiRISE-derived DEMs. Along each profile, we identified: (i) the locations of the edifice bases, defined as the break in slope relative to the surrounding terrain; (ii) the summit rims of central craters in the highest elevation point, or the top of the slope or summit peak in the case of flat-topped or peak-shaped edifices; and (iii) the elevation of the lowest point within the central crater (Fig. 1d). These measurements were then used to calculate the average morphological parameters of each edifice, including basal width (*W*_*CO*_), central crater width (*W*_*CR*_), their ratio, edifice height (*H*), depth of the central crater (*D*), slope, and circularity, defined as the ratio between the two basal widths. The same approach was applied to other conical-shaped edifices located in Aeolis Planum, the Aetheria region, Amazonis Planitia, Athabasca Valles, north of Olympus Mons, and the Tartarus region to provide morphological parameters for comparative analyses (Fig. 5).

To estimate the thickness of the lava flow underlying the phreatomagmatic edifices, we used the HiRISE-derived DEM to identify the flow margins, construct perpendicular topographic profiles (*n* = 118) across them, and determine the elevation of the lava flow base. As this lava flow advanced, it did not have complete areal coverage and thus left patches of the pre-existing surface visible. The elevation of these points was also measured and used. To reconstruct the pre-eruption surface, we applied the Inverse Distance Weighted (IDW) interpolation tool in ArcMap using all the identified base elevation points. By subtracting the current surface topography, represented by the HiRISE-derived DEM, from the reconstructed pre-eruption surface using the Minus tool in the ArcGIS Spatial Analyst toolbox, we obtained the residual elevation values that characterize the lava thickness distribution. To determine the lava flow thickness at the locations of the identified edifices, we extracted thickness values using the Extract Values to Points tool in ArcGIS.

### Crater counting

The small size of the phreatomagmatic edifices on Mars precludes direct crater-counting-based model age determination; therefore, relative stratigraphy was used. Since the documented edifices are directly superimposed on individual lava flows, these flows must have advanced over a water- or ice- logged layer shortly before edifice formation and thus provide the best approximation of their formation age. Moreover, in case when the age of the lava flow with superimposed edifices could not be determined, the adjacent and underlying lava flows were dated to determine a maximum age. Crater size distribution mapping for parts of the edifice-hosting lava flows that lack evidence of phreatic features, as well as for underlying lava flows was conducted on CTX images applying the ArcGIS plug-in, CraterTools2.1^92^. Crater statistics and derivation of crater model ages, including their errors, were carried out using the CraterStats II software^93^. To constrain the age of phreatomagmatic eruptions, fourteen lava flow surface areas, ranging from 13.6 to 97.6 km², were dated (Table S1). These lava flows host between 63 and 332 impact craters, of which 20 to 154 were specifically used for fitting Crater Size-Frequency Distributions (CSFDs). The minimum diameters of the mapped craters used for fitting the model age isochrones varied depending on the randomness analysis results^94^, which assesses the degree of clustering to evaluate potential contamination by secondary craters. The minimum crater diameters were always greater than 35–60 m, corresponding to approximately 7–12 pixels in CTX images and thus reliably identifiable^95,96^. This relatively small minimum diameter range has previously been used successfully for isochron fitting on other volcanic terrains based on CTX images^26,81,82,97,98^. In this study, we used ages provided based on the Hartmann^99^ chronology model and the Poisson timing analysis, which allowed us to avoid any bias related to the binning of crater size^100^. Alternative chronology systems have also been used to calculate the ages of lava flows, enabling cross-comparisons between studies. Differences in the resulting ages arise from variations in assumed cratering rates. Systems with higher cratering rates^101,102^ yield younger ages for the same crater density compared to the Hartmann^99^ system.

### CRISM data

Mineralogy determination was carried out using the Compact Reconnaissance Imaging Spectrometer for Mars (CRISM) infrared (IR; 1.0–2.6 μm) hyperspectral data at the Targeted Reduced Data Record (TRDR) level. One CRISM full resolution targeted (FRT) image with spatial resolution of 18 m px^-1^ (Table S3) was analyzed using the CRISM Analysis Toolkit (CAT) in ENVI/IDL software^103,104^. Raw CRISM data were downloaded from the NASA Planetary Data System and spectrally processed applying photometric and atmospheric corrections with the volcano-scan correction algorithm^105^.

For the initial identification of Regions of Interest (ROIs), a suite of CRISM spectral parameters representing reflectance at diagnostic wavelengths was compiled in the CRISM-delivered browse products. These parameters were combined and displayed as RGB components to highlight spatial variations in spectral characteristics associated with hydrated and hydroxylated minerals^46^. In this study, the HYS and PHY browse products highlight, in blue, areas exhibiting a hydration-related spectral signature consistent with the presence of hydrated mineral phases, including possible sulfates, carbonates, or hydrated silica, although these parameters are not uniquely diagnostic of specific mineral classes (Figure S2). The HYD browse product (Figure S2) highlights the presence of structurally bound or adsorbed water in minerals through hydration-related absorption features. Yellow-green pixels are consistent with hydrated mineral assemblages, including possible hydrated sulfates, but alone do not permit confident discrimination between mono- and polyhydrated sulfate phases or alternative hydrated mineral groups.

Consequently, as identified in the CRISM browse products, an average reflectance spectrum was extracted from the ROI, comprising several pixels (Fig. 3) located on the flank of the edifice. The averaged reflectance within the ROI (numerator) was normalized to spectrally neutral terrains (denominator) within the same detector columns to enhance spectral features (Figure S3). Owing to the high signal-to-noise ratio, the spectrally normalized spectrum for ROI#1 was smoothed using an adjacent-averaging method with a moving five-point window. Then to interpret the obtained mineral spectrum, we used the NASA RELAB laboratory spectra included in the CAT spectral libraries to compare the acquired spectrum with laboratory-derived spectra of kieserite (F1CC15), Mg-sulfate (799F366), gypsum (LASF41A), and alunite (F1CC08B)^46^. The normalized CRISM spectrum was plotted on the same diagram (Fig. 3) alongside the corresponding reference spectra, with an offset applied for clarity. The figures were prepared using ENVI/IDL products, visualized in ArcMap, and lastly compiled in CorelDRAW. All locations of the ROIs have been provided in the supplementary materials and deposited in the dedicated external repository for this study, available at the following link: 10.6084/m9.figshare.30468626.

## Supplementary information

Supplementary Material.

Supplementary Data 1.

## Data Availability

All original data used in this study are publicly available on the NASA Planetary Data System (PDS) at https://pds.nasa.gov/. The HiRISE-based DEMs used for topographic measurements and 3D visualizations have been produced using the Mars System of Information. The produced DEMs are available at the MarsSI repository and can be found together with CRISM spectral data and other Supplementary Data files in the online open-access repository at: 10.6084/m9.figshare.30468626.
