# Supplementary material for: Recent explosive lava-water interaction in Tharsis, Mars
Source: npj Space Explor. 2026 Apr 2;2(1):15. doi: 10.1038/s44453-026-00031-2 (PMC13046473; doi:10.1038/s44453-026-00031-2)
Supplement: Supplementary file 1 — Supplementary Material. [file 44453_2026_31_MOESM1_ESM.pdf]

SUPPLEMENTARY MATERIAL

Recent explosive lava-water interaction in Tharsis, Mars

Bartosz Pieterek[1*] and Thomas J. Jones[2*]

[1] Geohazard Research Unit, Institute of Geology, Adam Mickiewicz University in Poznań, Poznań, Poland

[2] Lancaster Environment Centre, Lancaster University, Lancaster, UK

*Corresponding authors: barpie@amu.edu.pl and thomas.jones@lancaster.ac.uk

SUPPLEMENTARY FIGURES

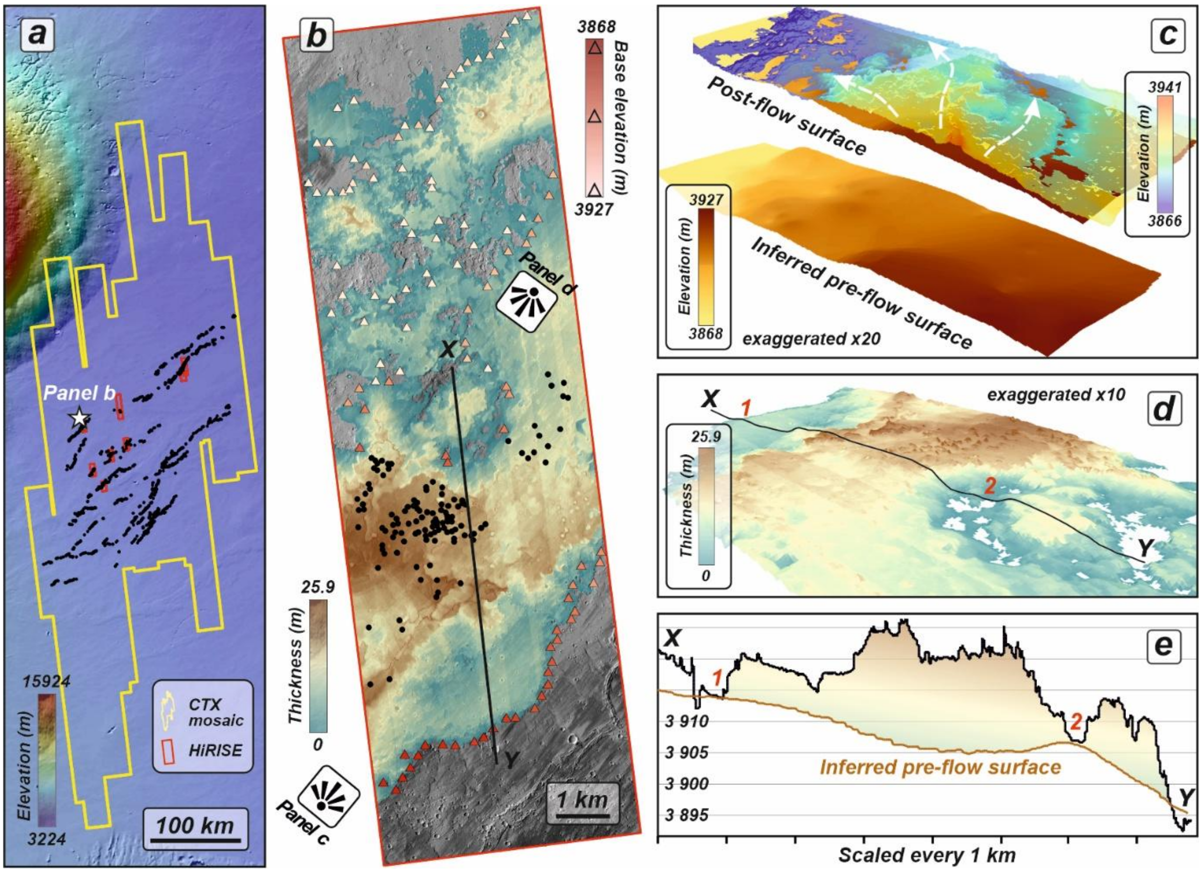

Fig. S1 Thickness measurements of a lava flow with superimposed rootless cones. (a) Topographic context map of the investigated area south of Ascraeus Mons, showing the distribution of rootless cones (black circles) and the datasets used in this figure. The basemap combines the MOLA shaded relief basemap (463 m px^{-1}) overlaid by the MOLA-HRSC DEM [1] (200 m px^{-1}). **(b)** HiRISE image partially overlaid by a lava flow thickness map derived from the corresponding DEM, which was generated from the stereo-pair HI_034172_1880 and HI_ 035596_1880. Superimposed rootless cones are marked by black circles. Triangles indicate locations where pre-flow surface elevations were determined using the HiRISE image and DEM-based topographic profiles. These points were used to interpolate the pre-flow surface using the Inverse Distance Weighted (IDW) tool in ArcMap. **(c)** 3D perspective views of the interpolated pre-flow surface and the current post-flow surface, vertically exaggerated 20 times. The HiRISE-derived DEM is draped over the reconstructed pre-flow surface.. White dashed arrows indicate the lava flow direction. **(d)** 3D view of lava flow thickness, vertically exaggerated 10 times. Base elevations were derived from the HiRISE DEM. The black solid line marks the location of the topographic profile (X–Y), while red numbers indicate depressions representing the pre-flow terrain, corresponding to the topographic profile shown in the next panel. **(e)** Topographic profiles of the pre-flow and current surfaces, derived from the respective elevation maps. The area has a general slope to the NNE, with a mean pre-flow surface slope of approximately 0.3°.

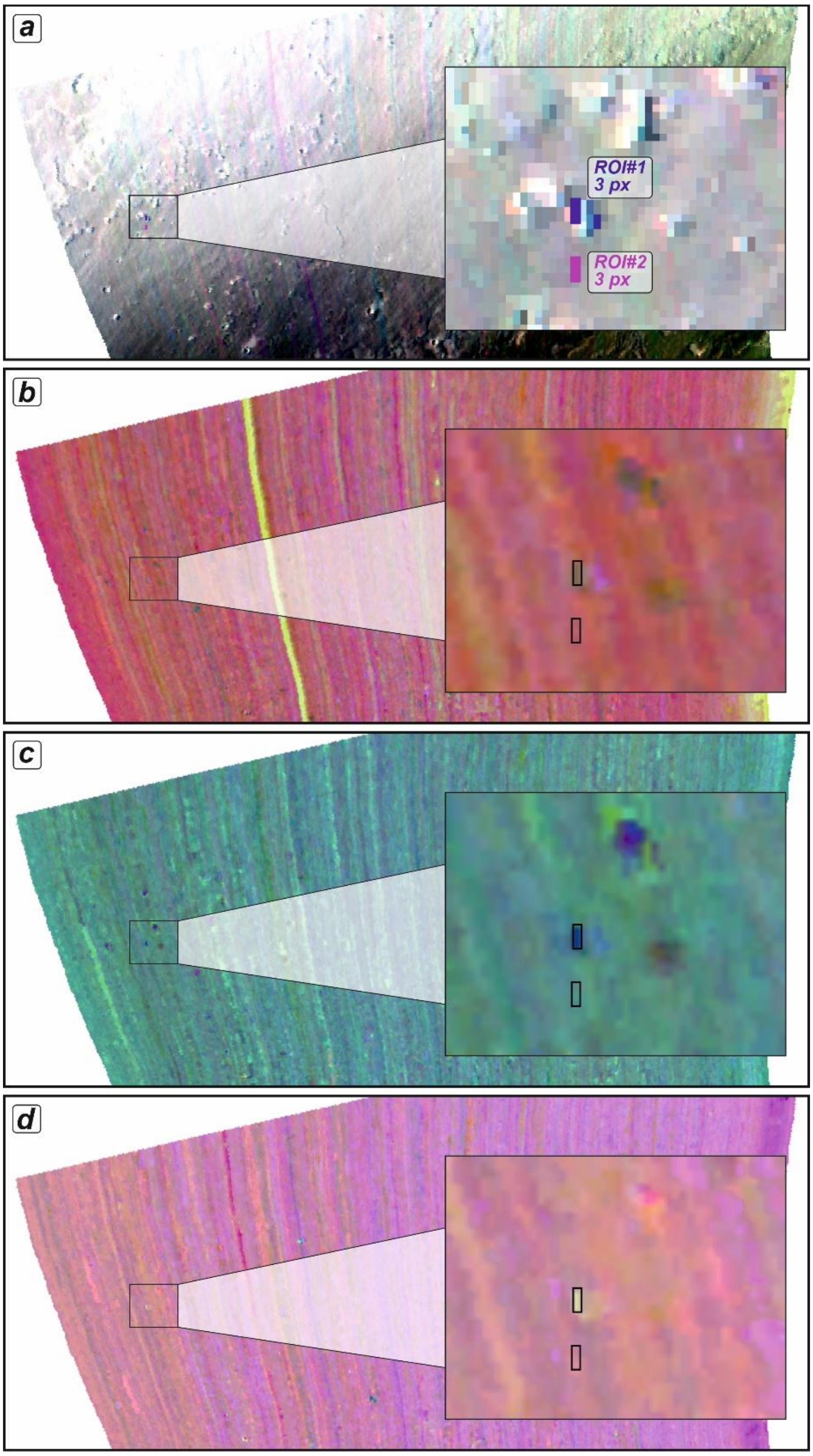

Fig. S2 Compilation of CRISM IR (infrared) browse products[2] illustrating the spatial distribution of hydrated mineral occurrences together with the region of interests (ROIs) selected for spectral analyses. (a) Map-projected Radiance/Reflectance (I/F) data with the locations of the ROIs. Across all panels, the zoomed-in images show the same area. **(b)** HYS (from "hydrated silica") browse product highlighting the occurrence of hydrated minerals (e.g., sulfates, clays, hydrated silica, carbonates, or water), shown in blue. **(c)** PHY (from "phyllosilicates") browse product related to hydroxylated minerals. Blue colors are indicative of hydrated phases (e.g., sulfates, hydrated silica, carbonates, or water ice). **(d)** HYD (from "hydrated mineralogy") browse product illustrating spectral information related to water bound within minerals. Yellow–green areas indicate the presence of monohydrated sulfates, characterized by a strong 2.1 μm absorption band and a weak 2.4 μm absorption band.

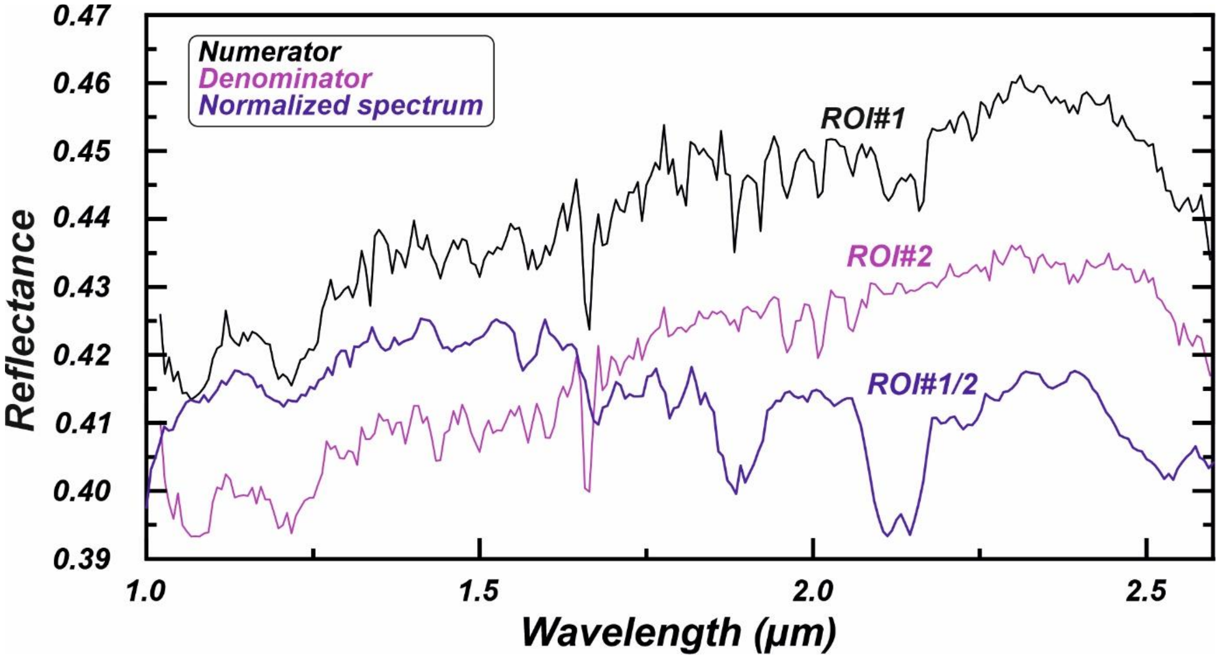

Fig. S3 Plot showing the spectral characteristics of the nominator (black; region of interest, ROI#1) and denominator (pink; ROI#2), together with the normalized spectrum (blue; ROI#1/2). The y-axis reflectance scale refers to the nominator and denominator spectra, whereas the normalized spectra are vertically offset for clarity.

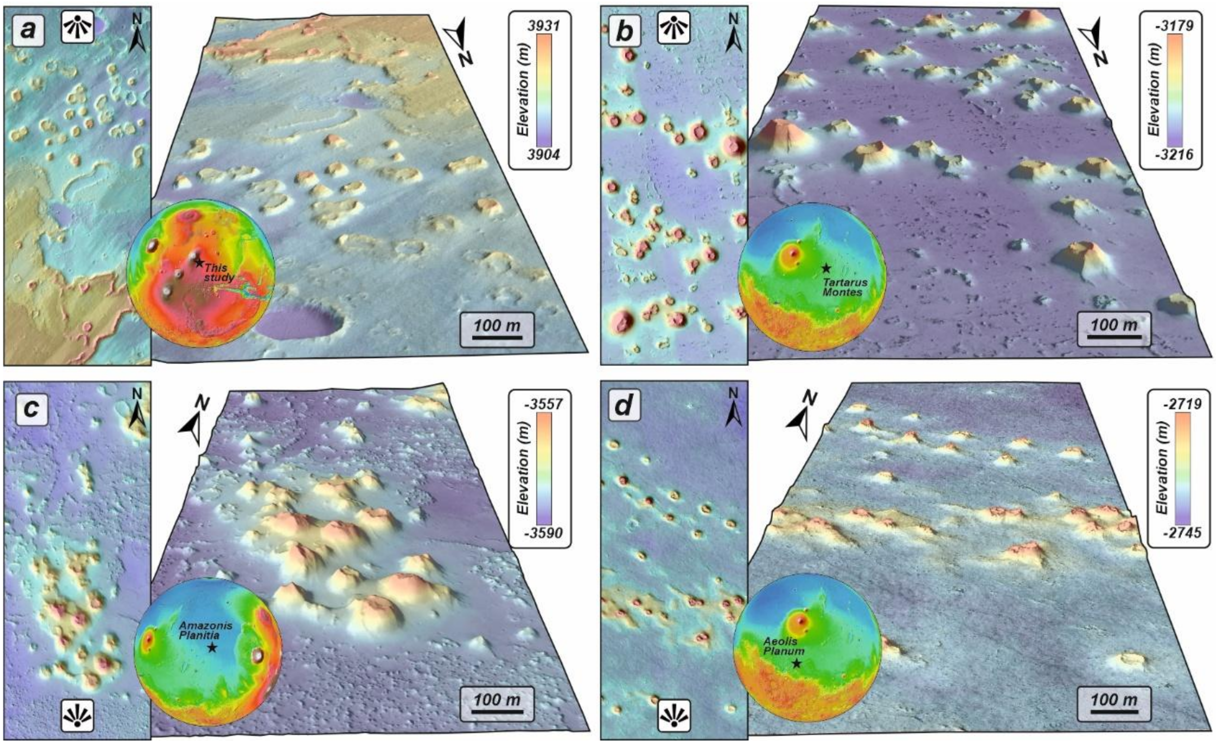

Fig. S4 Morphological comparison of selected fields across Mars hosting interpreted rootless cones. (a) Topographic map showing a cluster of rootless cones located south of Ascraeus Mons, investigated in this study. A 3D perspective view was generated using a combination of HiRISE imagery and the corresponding digital elevation model (DEM) to illustrate the morphology of the rootless cones superimposed on lava flows. The inset provides a context map of Mars, marking the location of the study area. Both the map and 3D view are derived from the HiRISE stereo pair HI_034172_1880 and HI_035596_1880, produced by MarsSI. The 3D view is vertically exaggerated by a factor of three to highlight the morphological characteristics of the terrain. **(b-d)** Analogous fields of rootless cones the interpreted by other studies in (b) Tartarus Montes[3], (c) Amazonis Planitia[4,5], and (d) Aeolis Planum[6]. Each panel includes a topographic map and a corresponding 3D perspective view of the region. Insets provide the Martian global context, indicating the locations of the study areas. The topographic maps and 3D views are derived from HiRISE stereo pairs: (b) HI_009675_2060 and HI_008528_2060, (c) HI_004202_1915 and HI_003701_1915, as well as (d) HI_003756_1825 and HI_004178_1825. The corresponding DEMs were obtained from the NASA/JPL/University of Arizona. Panels (b-d) are vertically exaggerated by a factor of two.

SUPPLEMENTARY TABLES

Table S1 The ages obtained by the surface crater counting of the lava flow units to constrain the age of occurrence of phreatomagmatic eruptions south of Ascraeus Mons and the formation age of the superimposed rootless cones. In this study, we applied three crater chronology systems—Hartmann and Neukum (2001)[7], Ivanov (2001)[8], and Hartmann (2005)[9] coupled with Michael (2013)[10]—to calculate reference model ages for the mapped units, thereby enabling direct comparison with previous studies.

Volcanic unit	Area (km^2)	Number of mapped craters	Number of craters used for fitting	Min. size of craters (m)	Max. size of craters (m)	Age (Ma)		
						Hartmann and Neukum, 2001	Ivanov, 2001	Hartmann, 2005 and Michael (2013)
R1	17.359	94	31	50	200	108 ± 19	128 ±23	178 ± 32
R3	13.653	63	32	35	150	46 ±8	54 ± 10	84 ±15
R4	97.589	108	67	50	200	42 ± 5	49 ± 6	69 ± 8
R5	32.953	146	62	40	200	56 ± 7	66 ± 8	98 ± 10
R6	30.976	185	85	40	200	82 ± 9	96 ± 10	142 ± 15
R8	16.236	113	57	40	200	104 ± 14	123 ± 16	182 ± 24
R9	23.376	190	20	60	200	96 ± 23	113 ± 27	154 ± 36
R10	15.222	131	24	50	200	95 ± 20	110 ± 20	158 ± 32
R12	54.002	168	84	50	250	94 ± 10	110 ± 12	154 ± 17
R13	51.106	332	154	40	300	89 ± 7	110 ± 8	155 ± 12
R14	27.258	122	35	50	110	83 ± 10	99 ± 20	140 ± 24
R15	14.453	114	60	40	200	123 ± 16	146 ± 19	215 ± 28
R16	39.284	199	85	50	400	130 ± 14	153 ± 17	213 ± 23

± is 1σ as age uncertainty based on the number of craters fitted.

Table S2 Summary of morphometric parameters derived from manual measurements based on HiRISE images and associated DEMs. Avg., mean average; 1 SD, one standard deviation.

Morphological parameters	*Basal width (m)*	*Crater width (m)*	*Height (m)*	*Crater/Basal width ratio*
Ascraeus Mons				
Avg.	95.53	42.66	3.83	0.42
1 SD	30.95	18.39	1.98	0.11
number of measurements (*n*)[1]	248	206	177	206
Amazonis Planitia (n = 34)				
Avg.	104.16	37.12	8.02	0.36
1 SD	30.47	13.41	2.70	0.07
N of Olympus Mons (n = 33)				
Avg.	392.37	124.52	28.73	0.32
1 SD	123.12	39.07	10.84	0.04
Athabasca Valles (n = 74)				
Avg.	53.15	19.52	n/d	0.36
1 SD	15.80	8.16	n/d	0.08
Aeolis Planum (n = 30)				
Avg.	167.92	54.42	19.89	0.32
1 SD	53.55	23.87	4.65	0.08
Tartarus Colles (n = 30)				
Avg.	145.03	47.39	14.20	0.33
1 SD	41.61	13.69	5.98	0.05
Hrad Vallis (n = 20)				
Avg.	131.11	38.52	5.36	0.29
1 SD	22.82	11.87	2.11	0.07

[1]This set of morphometric measurements integrates both HiRISE imagery and HiRISE-derived DEMs (see Methods for details).

Table S3 Details of the High Resolution Imaging Science Experiment (HiRISE) images and their corresponding stereo-pair DEMs, the Compact Reconnaissance Imaging Spectrometer for Mars (CRISM) spectral dataset, and the Context Camera (CTX) images used for this study. Dates are given in the format year-month-day.

Stereo-pair	Image ID	Imagery system	Center Latitude (°N)	Center Longitude (°E)	Scaled pixel grid (m px^{-1})	Acquisition date	Emission angle (°)	Incidence angle (°)
#1	ESP_034172_1880	HiRISE	7.691	258.156	0.50*	2013-11-10	6.9460	44.0267
	ESP_035596_1880	HiRISE	7.701	258.154	0.50*	2014-03-01	15.2862	49.5072
#2	ESP_033961_1870	HiRISE	7.136	258.655	0.50*	2013-10-24	9.6901	42.7739
	ESP_034462_1870	HiRISE	7.120	258.656	0.50*	2013-12-02	19.3848	44.5042
#3	ESP_034884_1890	HiRISE	8.781	260.009	0.50*	2014-01-04	26.7421	49.4416
	ESP_034818_1890	HiRISE	8.753	260.008	0.50*	2013-12-30	1.8665	47.3516
	ESP_017254_1885	HiRISE	8.573	259.979	0.25*	2010-04-01	11.9607	48.4291
	ESP_016832_1885	HiRISE	8.581	259.978	0.25*	2010-02-28	8.6288	44.8728
	ESP_038062_1870	HiRISE	6.864	258.325	0.50*	2014-09-09	9.1916	58.4076
	PSP_002209_1865	HiRISE	6.566	258.526	0.25*	2007-01-15	1.0046	54.3171
	ESP_037574_1875	HiRISE	7.334	258.939	0.50*	2014-08-02	1.8476	55.9079
	ESP_035174_1875	HiRISE	7.406	260.109	0.50*	2014-01-27	4.0255	49.1664
	FRT00016E46_07_DE165S	CRISM	8.610	259.988	18	2010-02-28	8.4820	44.9144
	D05_029214_1887	CTX	8.773	260.882	5.42	2012-10-19	0.34	55.67
	B18_016687_1887	CTX	8.639	259.132	5.46	2010-02-16	0.09	44.51
	P13_006178_1907	CTX	10.593	259.321	5.49	2007-11-20	0.10	36.89
	B18_016832_1895	CTX	9.386	259.873	5.57	2010-02-28	8.58	44.50
	B06_011808_1891	CTX	9.020	260.493	5.51	2009-02-01	5.28	59.81
	P13_005967_1901	CTX	10.009	260.075	5.56	2007-11-04	6.65	37.06
	F10_039592_1842	CTX	4.270	258.875	5.36	2015-01-06	0.92	52.28
	F23_044972_1866	CTX	6.617	259.931	5.35	2016-02-29	1.86	48.02
	U20_078956_1866	CTX	6.568	260.176	5.53	2023-05-31	8.69	51.39
	F02_036585_1880	CTX	7.953	258.569	6.15	2014-05-17	20.2	50.74
	U23_079800_1861	CTX	6.118	259.200	5.50	2023-08-05	7.95	54.28
	D05_029069_1867	CTX	6.667	259.696	5.40	2012-10-08	0.08	54.81
	F18_042928_1862	CTX	6.162	259.495	5.41	2016-02-08	0.33	43.08
	G01_018454_1854	CTX	5.372	257.466	5.42	2010-07-04	3.06	51.98
	F06_038062_1851	CTX	5.046	258.552	5.51	2014-09-09	9.15	58.18
	D22_035596_1877	CTX	7.680	258.130	5.82	2014-03-01	15.34	49.45
	P14_006600_1881	CTX	8.101	258.009	5.47	2007-12-23	6.26	37.11
	P13_006244_1869	CTX	6.879	257.756	5.43	2007-11-25	0.10	35.79
	D18_034172_1877	CTX	7.680	258.165	5.50	2013-11-10	6.92	44.00
	B17_016120_1861	CTX	6.044	258.915	5.39	2010-01-03	0.10	41.31
	P03_002209_1864	CTX	6.311	258.550	5.41	2007-01-15	1.04	54.33
	G22_026814_1883	CTX	8.238	258.885	5.43	2012-04-15	0.79	48.82
	D07_029847_1883	CTX	8.283	258.665	5.41	2012-12-08	0.09	57.10
	G22_026669_1828	CTX	2.861	258.126	5.36	2012-04-04	0.09	51.38
	U11_075435_1866	CTX	6.568	258.126	5.52	2022-08-30	9.01	47.98
	P20_009039_1834	CTX	3.439	258.545	5.36	2008-06-30	0.09	53.05
	U09_074512_1834	CTX	3.430	257.912	5.33	2022-06-19	0.08	55.33

*HiRISE resolution is given for map-projected images.

REFERENCES

1. Fergason, R. L., Hare, T. M. & Laura, J. HRSC and MOLA blended digital elevation model at 200m v2, astrogeology PDS annex. *US Geological Survey* (2018).

2. Viviano, C. E. *et al.* Revised CRISM spectral parameters and summary products based on the currently detected mineral diversity on Mars. *J. Geophys. Res. Planets* 119, 1403–1431 (2014).

3. Hamilton, C. W., Fagents, S. A. & Thordarson, T. Lava-ground ice interactions in Elysium Planitia, Mars: Geomorphological and geospatial analysis of the Tartarus Colles cone groups. *J. Geophys. Res. Planets* 116, E03004 (2011).

4. Lanagan, P. D., McEwen, A. S., Keszthelyi, L. P. & Thordarson, T. Rootless cones on Mars indicating the presence of shallow equatorial ground ice in recent times. *Geophys. Res. Lett.* 28, 2365–2367 (2001).

5. Bruno, B. C., Fagents, S. A., Thordarson, T., Baloga, S. M. & Pilger, E. Clustering within rootless cone groups on Iceland and Mars: Effect of nonrandom processes. *J. Geophys. Res. Planets* 109, E07009 (2004).

6. Lanz, J. K. & Saric, M. B. Cone fields in SW Elysium Planitia: Hydrothermal venting on Mars? *Journal of Geophysical Research E: Planets* 114, E02008 (2009).

7. Hartmann, W. K. & Neukum, G. Cratering choronology and the evolution of Mars. *Space Sci. Rev.* 96, 165–194 (2001).

8. Ivanov, B. A. Mars/Moon cratering rate ratio estimates. *Space Sci. Rev.* 96, 87–104 (2001).

9. Hartmann, W. K. Martian cratering 8: Isochron refinement and the chronology of Mars. *Icarus* 174, 294–320 (2005).

10. Michael, G. G. Planetary surface dating from crater size-frequency distribution measurements: Multiple resurfacing episodes and differential isochron fitting. *Icarus* 226, 885–890 (2013).

CAPTION FOR SUPPLEMENTARY DATA 1

Dataset of morphological measurements for the identified rootless cones located south of Ascraeus Mons, Tharsis. The table includes feature coordinates, mapped source image information, and derived morphometric parameters. All measurements were obtained along N–S and W–E topographic profiles using High Resolution Imaging Science Experiment (HiRISE)–derived digital elevation models (DEMs). For cones not covered by HiRISE stereo-pair acquisitions, parameters requiring elevation data could not be measured; therefore, only basal and summit crater widths are reported. For rootless cones identified solely from Context Camera (CTX) images, morphological characteristics are unavailable. Summary statistics for all morphological parameters are provided at the bottom of the table.
